# Health after childbirth

**DOI:** 10.1016/j.crwh.2024.e00629

**Published:** 2024-06-11

**Authors:** G. Justus Hofmeyr

**Affiliations:** a Departments of Obstetrics and Gynaecology, University of Botswana, Botswana; b Walter Sisulu University and University of the Witwatersrand, South Africa

**Keywords:** Childbirth, Evolution, Adverse outcomes, Clinical environment, Mitigating strategies

Giving birth comes at a cost. A recent review catalogues the litany of health problems which may complicate the days, months and years following childbirth, and the lack of attention paid to this suffering [1].

It is difficult to understand why a normal biological process such as birth should have adverse health consequences, unless viewed from an evolutionary perspective. Natural selection would be expected to drive the evolution of biological processes which favour the next generation. Teleologically, the fetus and placenta are programmed to flourish, placing considerable demands on their host. Central to this inequity is the attribute which distinguishes us most emphatically from other species, the human brain. Evolution of such a metabolically avaricious organ has required extreme biological adaptations [2]. These include high-volume blood flow to the placenta, placing demands on the cardiovascular system; remodelling of placental bed arterioles to wide, non-contracting vessels with the potential for life-threatening blood loss after placental separation; evolution of placental factors which prevent endothelialisation of the intervillous space, creating the uniquely efficient haemochorial placental structure but also the potential for devastating generalised endothelial damage known as pre-eclampsia [3]; enhanced immune tolerance, increasing the risk of infection, for example with HIV; enhanced coagulation, with the potential for thromboembolism; hormonal adaptations such as prolactin surges with the potential to disrupt myocardial contractility, known as peripartum cardiomyopathy; emotional changes linked to postpartum depression or psychosis; a fetal brain size close to the limits of pelvic capacity, which may result in damage to pelvic structures, in turn causing urinary or fecal incontinence or dyspareunia, or which may obstruct birth altogether; and many more adverse consequences.

What can we, as health workers, and society in general, do to mitigate the adverse consequences of childbirth? Vogel and colleagues have comprehensively detailed the need for greater acknowledgement, screening, prevention, care, advocacy and research into longer-term consequences of childbirth, particularly in low- and middle-income countries [1]. Here we shall focus on a few key strategies.

The tragedy of morbidity or mortality following childbirth becomes unbearable when the pregnancy was undesired. First and foremost, ensuring access to free, high-quality family-planning services for *everyone* needs to be at the top of every developmental and national agenda.

Secondly, we need to consider the extent to which the clinical care we provide may contribute to poor childbirth outcomes. There is considerable observational and experimental evidence suggesting that stress may cause poor pregnancy and childbirth outcomes, and this may include stress related to the clinical environment. In a randomized trial in Zimbabwe, routine admission of those with twin pregnancy for bedrest in hospital for the purpose of reducing the risk of preterm birth was actually associated with significantly more preterm births than in the control group, plausibly an effect of the stress of hospitalisation [4]. A mindfulness-based stress-reduction programme has been reported to reduce adverse pregnancy outcomes [5]. In the late 1980s we tested the hypothesis that the clinical environment might cause adverse childbirth outcomes, and that this effect might be ameliorated by human companionship. We randomly allocated consented first-time parturients to routine care during labour or the presence of a community volunteer chosen on the basis of her capacity to express love, and asked to do nothing more than to provide comfort, reassurance and praise. Compared with the control group, those receiving this human interaction showed significant reductions in anxiety, use of analgesia, and diastolic blood pressure; and improved sense of coping with labour and breastfeeding [6]. In the longer term, there was a remarkable reduction in postnatal depression and improved self-esteem and confidence in childcaring competence [7]. Review of 26 randomized trials found that continuous support in labour was associated with a 25% reduction in caesarean birth and 38% reduction in low Apgar scores, among many benefits [8]. Implementation of childbirth companion programmes in low- and middle-income countries has proved exceptionally difficult, but the barriers are not insurmountable [9].

The World Health Organization (WHO) has led the call for a revised approach to care during childbirth *for a positive birth experience*. At Princess Marina Hospital in Botswana, we have institutionalised the care principles of the WHO Labour Care Guide [10] with the mnemonic COPE: Companions; Oral fluids; Pain relief; and, most critically, Eliminate the supine position for birth. To institutionalize recent advances in third-stage labour care, we use the mnemonic BOND: Baby skin to skin; Oxytocic; iNitiate blood loss monitoring; and Delay cord clamping (especially for preterm births).

For those with means, caesarean birth by choice has increasingly been viewed as a way of avoiding at least the pelvic structure damage of childbirth, but at considerable cost: increased maternal mortality; more neonatal respiratory morbidity and high care admissions; more childhood illness; more subsequent infertility; and risks in future pregnancies such as placenta praevia spectrum and uterine rupture. An unsatisfactory solution, and one not available to the vast majority. Rather, we need to advocate for attention to neglected suffering, and minimize adverse impacts of childbirth by taking seriously and actually implementing the many proven effective simple and inexpensive changes to the way we provide care, and undertaking more research addressing the neglected adverse consequences of childbirth.
